# Atteinte ganglionnaire au cours du myélome multiple

**DOI:** 10.11604/pamj.2015.21.224.7402

**Published:** 2015-07-30

**Authors:** Amira Atig, Neirouz Ghannouchi Jaafoura

**Affiliations:** 1 Service de Médecine Interne, CHU Farhat Hached, Sousse, Tunisie

**Keywords:** Myélome multiple, localisation ganglionnaire, adénopathie, multiple myeloma, lymph node localization, lymphadenopathy

## Image en medicine

Mr A.K, âgé de 70 ans, est suivi depuis 2 ans pour un myélome multiple type Ig A kappa stade III A selon la classification de Salmon et Durie. Le patient est hospitalisé pour une fièvre, une douleur mécanique de l’épaule gauche et une altération de l’état général évoluant depuis 3 semaines. L'examen physique à l'admission, objective une adénopathie sus-claviculaire gauche de 2 cm, mobile, ferme et indolore sans signes inflammatoires en regard. Il n'y a ni hépatomégalie ni splénomégalie et le reste de l'examen physique est sans anomalies. A la biologie, on note une anémie normochrome normocytaire à 8,4g/dl, une lymphopénie à 500 éléments/mm3, une vitesse de sédimentation à 26 mm à la première heure, une calcémie à 2,5 mmol/l et une créatininémie à 79 µmol/l. L’électrophorèse des protides révèle un pic monoclonal en béta 2 à 21,89g/l. La radio de thorax montre une image lytique au niveau du tiers externe de la clavicule gauche (A). Une biopsie du ganglion sus-claviculaire est réalisée et l'examen anatomopathologique trouve une prolifération plasmocytaire (B). A l’étude immuno-histochimique, les cellules tumorales expriment le CD 138, confirmant la nature plasmocytaire de la prolifération (C). L'atteinte ganglionnaire au cours du myélome multiple est rare mais peut toucher toutes les aires ganglionnaires. Elle survient souvent au cours de l’évolution de la maladie.

**Figure 1:**
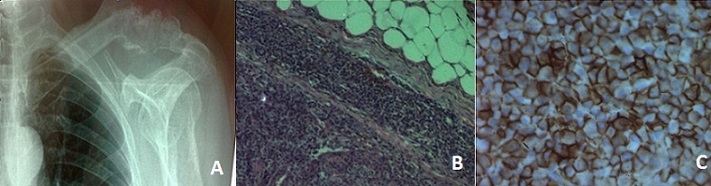
(A): radio thorax de face: image lytique au niveau du tiers externe de la clavicule gauche. (B): coupe ganglionnaire montrant une architecture éffacée et remplacée par une prolifération cellulaire diffuse sans effraction capsulaire. (C): étude immunohistochimique: les cellules tumorales expriment de façon diffuse et intense le CD 138 en faveur de la nature plasmocytaire

